# Comparative Study between Sequential Automatic and Manual Home Respiratory Polygraphy Scoring Using a Three-Channel Device: Impact of the Manual Editing of Events to Identify Severe Obstructive Sleep Apnea

**DOI:** 10.1155/2015/314534

**Published:** 2015-08-12

**Authors:** Glenda Ernst, Martín Bosio, Alejandro Salvado, Facundo Nogueira, Carlos Nigro, Eduardo Borsini

**Affiliations:** ^1^Respiratory Medicine Unit, British Hospital, Perdriel 74, C1280AEB Buenos Aires, Argentina; ^2^Respiratory Medicine Unit, Hospital Clinics, Cordoba Avenue 2351, C1120AAF Buenos Aires, Argentina; ^3^Respiratory Medicine Unit, Hospital Alemán, Pueyrredón Avenue 1640, C1118AAT Buenos Aires, Argentina

## Abstract

*Objective*. According to current guidelines, autoscoring of respiratory events in respiratory polygraphy requires manual scoring. The aim of this study was to evaluate the agreement between automatic analysis and manual scoring to identify patients with suspected OSA.* Methods*. This retrospective study analyzed 791 records from respiratory polygraphy (RP) performed at home. The association grade between automatic scoring and manual scoring was evaluated using Kappa coefficient and the agreement using Bland and Altman test and intraclass correlation coefficient (CCI). To determine the accuracy in the identification of AHI ≥ 30 eV/h, the ROC curve analysis was used.* Results*. The population analyzed consisted of 493 male (62.3%) and 298 female patients, with an average age of 54.7 ± 14.20 years and BMI of 32.7 ± 8.21 kg/m^2^. There was no significant difference between automatic and manual apnea/hypopnea indexes (aAHI, mAHI): aAHI 17.25 (SD: 17.42) versus mAHI 21.20 ± 7.96 (*p*; NS). The agreement between mAHI and aAHI to AHI ≥ 30 was 94%, with a Kappa coefficient of 0.83 (*p* < 0.001) and a CCI of 0.83. The AUC-ROC, sensitivity, and specificity were 0.99 (CI 95%: 0.98-0.99, *p* < 0.001), 86% (CI 95%: 78.7–91.4), and 97% (CI 95%: 96–98.3), respectively.* Conclusions*. We observed good agreement between automatic scoring and sequential manual scoring to identify subjects with AHI ≥ 30 eV/h.

## 1. Introduction

The study of sleeping disorders to determine diagnosis of obstructive sleep apnea (OSA) has been categorized in four levels of complexity according to the Standards of Practice Committee of the American Sleep Disorder Association since 1994 [1]. Moreover, the American Academy of Sleep Medicine (AASM) recommends level III devices (airflow, oximetry, and respiratory effort) when portable monitors are used to identify patients with suspected OSA [2]. It has been estimated that 75% of patients with suspected diagnosis of OSA can be handled with this type of device [3, 4].

Currently published guidelines for outpatient diagnosis of OSA in adults by respiratory polygraphy recommend that a properly trained operator performs manual analysis of respiratory signals because of its greater diagnostic accuracy compared to automated analysis [2, 5]. These recommendations were based on analysis of studies conducted in the sleep laboratory where some devices available in the market were compared with polysomnography in the sleep lab [2, 6–8]. Extrapolation of these observations to other respiratory polygraphs is questionable since the technology and algorithms for automatic analysis vary according to each device. Furthermore, few studies have compared the autoscoring versus manual scoring of the portable cardiorespiratory monitoring devices performed in the patient's home [9, 10]. On the other hand, the performance of the autoscoring could be related to the severity of OSA [11]. Thus, the aim of this study was to compare the agreement degree between automatic and manual analysis of a self-administered respiratory polygraph in a large population of subjects with suspected OSA.

## 2. Materials and Methods

### 2.1. Study Design

We conducted a retrospective study. The protocol was approved by the ethical committee of “British Hospital” in accordance with the ethical standards of Helsinki Declaration. Informed consent was obtained from all patients and control subjects.

We reviewed records from 791 patients with suspected respiratory sleep disorders (snoring, sleep apnea, or diurnal somnolence) from the Respiratory Medicine Unit at British Hospital between January 2010 and January 2013. We excluded patients with chronic heart failure and neuromuscular disease, subjects that used oxygen, CPAP, or treatment with other techniques of noninvasive ventilation, and those records with less than 4 hours valid to manual editing.

### 2.2. Measurements

All patients completed the Epworth Sleepiness Scale (ESS) and the Berlin questionnaire and underwent in-home respiratory polygraphy (RP). RP was taken by the ApneaLink Plus device (ResMed Australia) that included nasal pressure cannula, respiratory effort band, and oximetry. The ApneaLink Plus device is battery operated and has a sampling rate of 100 Hz and a 16-bit signal processor. The internal memory storage is 15 MB, which allows for approximately 10-hour data collection. All patients were trained on the use of the device which included a demonstration of its use and were given a copy with iconographic instructions about the procedure. All records were downloaded, analyzed automatically using the ApneaLink software (version 9.0), and finally edited using manual rules in sequential form.

### 2.3. ApneaLink Plus Analysis

#### 2.3.1. Automatic Scoring

The software reports apneas, hypopneas, flow limitation, snoring, and the apnea/hypopnea index (number of apneas plus hypopneas per hour of evaluation period). The evaluation period is the total recording time minus the time not considered in the analysis (invalid data, missing data, start of evaluation, end of evaluation, and too small signal). The ApneaLink Plus default settings for apneas and hypopneas were used in this study. Apnea was defined as a decrease in airflow by 80% from baseline for at least 10 s. The ApneaLink default maximum apnea duration was set at 80 s. Hypopnea was defined as a decrease in airflow by ≥50% from baseline for at least 10 s plus oxygen desaturation ≥3%. The ApneaLink default maximum hypopnea duration was set at 100 s. The automatic apnea/hypopnea index (AHI-a) was calculated as the number of apneas/hypopneas per hour of evaluation period.

#### 2.3.2. Manual Scoring

Once the ApneaLink software had carried out the automatic analysis, the results were revised in 3- or 5-minute epochs and, when appropriate, manually corrected by a trained physician. If required, the operator could edit or delete events or insert new ones. Likewise, it was possible to include or exclude sectors of the recording for its analysis. Apnea was defined as a decrease in airflow by 80% from baseline for ≥10 s and hypopnea was considered when a reduction in the airflow by 50% of baseline was observed for at least ≥10 s plus oxygen desaturation ≥3% [12]. The manual apnea/hypopnea index was calculated as the number of apneas/hypopneas per hour of evaluation period (mAHI). Patients were classified as normal (mAHI < 5 eV/h), mild (5 ≤ mAHI < 15), moderate (15 ≤ mAHI < 30), and severe OSA (mAHI ≥ 30 eV/h).

### 2.4. Statistical Analysis

We used “*t*-test” for independent samples to compare continuous variables and *χ* ^2^ using Fisher test to compare their proportions. To determine if the data had normal distribution, we used Kolmogorov-Smirnov test. All variables with normal distribution were expressed as average and standard deviation, and the variables without normal distribution were expressed as median and percentiles (25–75%). The association grade between automatic and manual scoring of ApneaLink Plus for the AHI was evaluated using Kappa coefficient. The grade of agreement between automatic and manual AHI was evaluated using Bland and Altman test and intraclass variation coefficient. To determine the accuracy between both identifications (aAHI ≥ 30 eV/h and mAHI ≥ 30 eV/h), we used ROC curves. All statistical analysis was performed using STATA 10.0 software and GraphPad Prism-5 software.

## 3. Results and Discussion

### 3.1. Demographic Characteristics of the Studied Population

Of the 791 patients who had in-home RP, 70 cases needed to perform a second record so the rate of repetition was 8.8%. There were 493 male (62.3%) and 298 female patients. The mean age and BMI were 54.7 ± 14.20 years and 32.7 ± 8.21 kg/m^2^, respectively. The 88% showed high risk of OSA in Berlin questionnaire and the mean Epworth was 8.4 ± 4.79 (Table 1).

### 3.2. Respiratory Polygraphy

**Figure 1:**
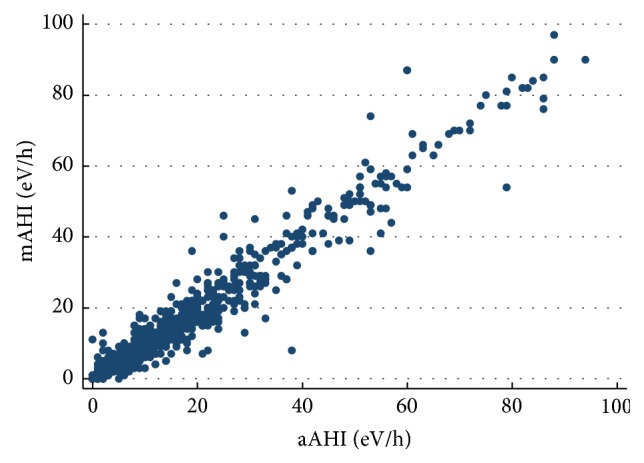
Relationship between mAHI and aAHI.

Studies analyzed had a mean total recording time (TRT) of 350 ± 142 minutes. There was no significant difference between the values of mAHI and aAHI (16.87 ± 17.71* versus *17.25 ± 17.42, *p*; NS) and manual and automatic oxygen desaturation index (20.31 ± 17.96* versus *21.20 ± 17.96, resp.) (Table 2(a)). A strong relationship between mAHI and aAHI in all population analyzed was shown (Spearman correlation coefficient *r* = 0.95 (95% confidence interval: 0.95-0.96), *p* < 0.0001 (Figure 1)).

We observed that automatic scoring identified 137 patients with severe OSA (AHI ≥ 30 eV/h) while manual scoring found 128 (Table 2(b)). But, in 81 cases, both automatic and manual scoring coincided in the diagnosis of severe OSA. We observed underestimation by automatic scoring because forty-seven patients were classified as moderate OSA (aAHI ≥ 15 and <30) while manual scoring found severe OSA in these same patients (mAHI ≥ 30). However, no patients with aAHI ≥ 30 were classified as mAHI < 15.

**Figure 2:**
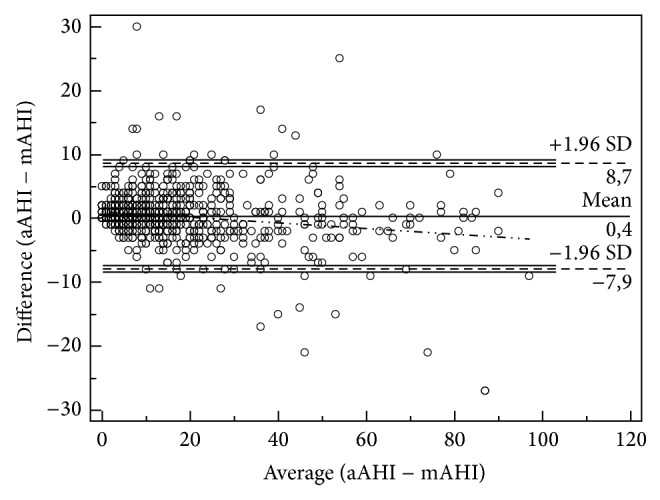
The distribution of both mAHI and aAHI.

The strength of agreement between mAHI and aAHI to detect patients with severe OSA (AHI ≥ 30) got a Kappa coefficient of 0.83 (*p* = 0.00001). The concordance between both scorings is showed in Figure 2. We also measured the intraclass correlation coefficient (CCI). We found a good ICC (0.83) when all patients were analyzed (*n*, 791). Moreover, when analyzed separately, patients with AHI ≥ 30 had an ICC of 0.91.

**Figure 3:**
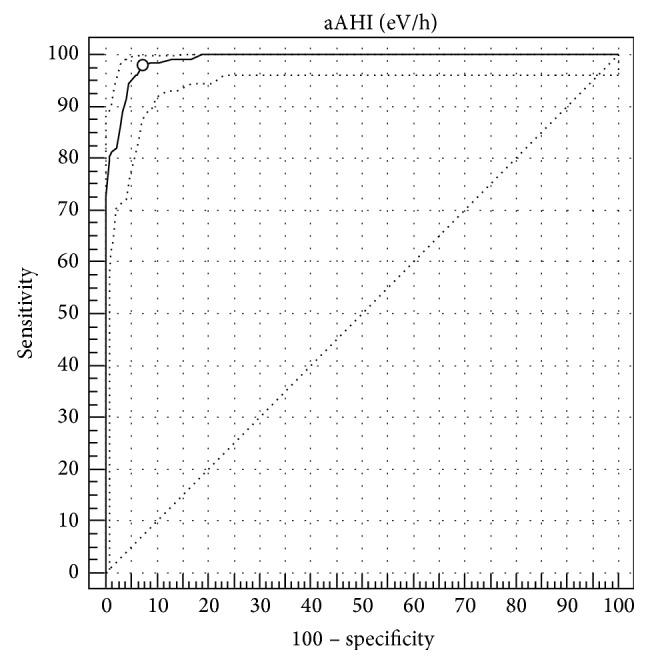
Comparison between mAHI and aAHI (≥30 eV/h). AUCs-ROC.

The comparison between aAHI and mAHI is shown in the ROC curve (AUC-ROC: 0.99; CI 95%: 0.981–0.996; *p* < 0.0001) for AHI ≥ 30 with sensitivity of 85.9 (CI 95%: 78.7–91.4) and specificity of 97.1 (CI 95%: 95.6–98.3); LR+ of 29.9 (CI 95%: 19.1–47.0); LR− of 0.14 (CI 95%: 0.09–0.2) (Figure 3). Similar analyses between aAHI and mAHI for AHI > 15 showed AUC-ROC 0.98 (CI 95%: 0.97-0.98; *p* < 0.001) with sensitivity of 96.2 and specificity of 89.3; LR+ of 8.95; LR− of 0.04; and for AHI > 5, we found AUC-ROC 0.97 (CI 95%: 0.96–0.98; *p* < 0.001) with sensitivity of 97.9 and specificity of 75.7; LR+ of 4.05; LR− of 0.03.

We indicated CPAP therapy to 254 patients (32.11%) based on final result of mAHI. Automatic scoring classified them as mild OSA: 50 patients (19.69%), moderate OSA: 129 (50.79%), and severe OSA: 75 (29.53%) (Table 3). Moreover, forty-seven patients misclassified (underestimated) as moderate OSA by the aAHI were treated with CPAP.

## 4. Discussion

The high prevalence of OSA imposes the need to find effective diagnostic strategies with simple and fast tests to identify patients that require treatments due to their high cardiometabolic risk. This is the main reason why home respiratory polygraphy is currently an attractive test that could provide a diagnostic approach [2–4]. Currently, the AASM has suggested performing manual scoring of the recordings since the automatic system presents limitations. However, this task takes time and requires trained staff.

Recommendations about systematic manual scoring were based in part on trials with limited number of patients and even with level IV devices [2, 13]. Differences between aAHI and mAHI using polysomnography versus level III devices have been previously described. Dingli et al. using RP and PSG in lab with synchronous recording found AHI differences of 3 ± 9 events/hour [7]. Other studies using different algorithms show that the automatic data detected 9–20 fewer events/hour less than the manual scoring [6, 8]. In practice, manual scoring of portable devices (six studies) compared with polysomnography during the same night in hospital had high pooled sensitivity of 0.93 (CI 95%: 0.89–0.97) and high specificity of 0.92 (CI 95%: 0.87–0.96). A systematic revision by the Nordic Project found no heterogeneity, even though 6 different portable equipment brands were used [5].

The automatic systems which are investigated identified most patients with obstructive sleep apnea, but specificity was low due to a high number of false positive results in the study by Dingli et al. using the Embletta automatic scoring system.

Reichert and his colleagues have found 95% of sensitivity and 91% of specificity to the manual scoring using cut-off 15 eV/h, using a level III system (Novasom) [14]. In accordance with them, we observed similar findings using IAH > 30. In a systematic revision, three different automatic systems were used [5, 7]; nevertheless, the results were not applicable to one another [5, 7].

Tiihonen and his colleagues have reported that a high number of mild to moderate OSA patients received false negative diagnosis using automatic scoring records from two home devices (Venla and Embletta) compared with manual scoring [15]. However, the effectiveness between sequential automatic and manual records using home respiratory polygraphy has recently been described as a cost-effective alternative to polysomnography for patients with severe and moderate OSA [16]. What is more, Yin and coworkers analyzed aAHI/mAHI using level III devices in a small number of patients and found an agreement of 61.4% with a Kappa coefficient of 0.554 [17]. However, it has been demonstrated that some devices are using similar diagnostic algorithms with high agreement between automatic and manual scoring, especially in patients with AHI > 25 [11, 18]. In our work, we found similar results in order to identify patients with moderate and severe OSA.

In this study, we evaluated if the automatic scoring using a self-administered in-home RP would be able to diagnose patients with elevated AHI (severe OSA). Using ApneaLink Plus device, we got high sensitivity for identified patients as severe OSA, based only on automatic scoring. This device was self-placed and this strategy can be useful in countries like ours, with very big distances to diagnostics centers and limited resources. ApneaLink Plus is a basic device with few channels in comparison with polygraphic systems used in other similar experiences, and probably for this reason it is less expensive. [5–8, 15, 17]. However, the effort signal has less quality in relation to other devices with RIP and has no thermistor or body position.

This work, which was retrospective with typical limitations about this type of analysis, did not study event classification; however, the profile of our patients was obstructive. It has been previously described as an underestimation of hypopnea events with RP and it could be affected by hypopnea criteria used [17]. We found good level of agreement and discrimination between automatic and manual scoring in the identification of patients with AHI ≥ 30 eV/h using ROC curves. This finding may gain importance in centers with waiting lists and represent a simplified strategy useful in primary care.

Decisions about the treatment with CPAP were taken with RP results and medical history. Some of the current recommendations suggest considering CPAP therapy for severe or moderate cases with associated symptoms or comorbidities [3, 18]. In our experience, patients misclassified as moderate OSA by the automatic scoring finally received CPAP recommendations.

To conclude, we suggest clinical utility of the automatic scoring when AHI is elevated (≥30) and the quality of the recordings is optimal. This finding could contribute to reduce time and human resources in the manual editing of level III RP devices.

## Figures and Tables

**Table 1:** Demographic characteristics of the population studied. Values are expressed as media and standard deviation.

Variable	Value
*n*	791
Male	493 (62.3%)
Age (years)	54.7 ± 14.20
BMI (kg/m^2^)	32.7 ± 8.21
ESS	8.37 ± 4.79
High risk of Berlin questionnaire	88.37%

*n*: number of patients; BMI: body mass index; ESS: Epworth Sleepiness Scale.

**(a):** 

	AHI (eV/h)	ODI (eV/h)
Manual	16.87 ± 17.71	20.31 ± 17.96
Automatic	17.25 ± 17.42	21.20 ± 17.96

**(b):** 

	aAHI	mAHI
<5	173	176
≥5 and <15	288	296
≥15 and <30	193	191
≥30	137	128

aAHI: automatic scoring of apneas hypopneas index; mAHI: manual scoring of apneas hypopneas index; ODI: oxygen desaturation index.

**Table 3:** Intention to treat with CPAP according to mAHI. Relationship with aAHI.

	No-CPAP	CPAP	All patients
<15	487	50	537
	90.69%	9.31%	67.89%

15 to 30	44	129	173
	25.43%	74.57%	21.87%

>30	6	75	81
	7.41%	92.59%	10.24%

All patients	537	254	791
	67.89%	32.11%	100%

CPAP: continuous airway pressure.
